# Human Umbilical Cord-Derived Mesenchymal Stem Cells Alleviate Acute Lung Injury Caused by Severe Burn via Secreting TSG-6 and Inhibiting Inflammatory Response

**DOI:** 10.1155/2022/8661689

**Published:** 2022-02-18

**Authors:** Xiaohong Hu, Lingying Liu, Yu Wang, Yonghui Yu, Zhongyuan Li, Yanan Liu, Jiake Chai

**Affiliations:** ^1^Department of Pediatrics in the Fourth Medical Center, Chinese PLA General Hospital, Beijing 100048, China; ^2^Department of Nutrition in the Fourth Medical Center, Chinese PLA General Hospital, Beijing 100048, China; ^3^Beijing Engineering and Technology Research Center of Food Additives, Beijing Technology and Business University, Beijing 100048, China; ^4^Department of Burn & Plastic Surgery in the Fourth Medical Center, Chinese PLA General Hospital, Beijing 100048, China

## Abstract

**Objectives:**

To investigate whether hUC-MSCs attenuated severe burn-induced ALI and the effects were based on TSG-6 secreted from hUC-MSCs.

**Method:**

A rat model was established and evaluated as follows: cytokine expression was measured by ELISA, and both inflammatory cell infiltration and lung injury were assessed by immunohistochemistry assay.

**Results:**

In vitro, TSG-6 levels in serum from the burn group were significantly increased compared with those from the sham group. In vivo, TSG-6 levels of lung tissues and serum in the burn+hUC-MSC group were significantly increased compared with those in the burn group. Both in lung tissues and in serum, increased levels of proinflammatory cytokines (TNF-*α*, IL-1*β*, and IL-6) were remarkably decreased, but the anti-inflammatory cytokine IL-10 increased after hUC-MSC administration (*p* < 0.05). These significant positive effects after hUC-MSC transplantation did not occur in the burn+siTSG-6 group.

**Conclusion:**

The intratracheal implantation of hUC-MSCs has been an effective treatment for severe burn-induced ALI via promoting TSG-6 secretion and inhibiting inflammatory reaction in lung tissue.

## 1. Introduction

Acute lung injury (ALI) and its severe manifestation acute respiratory distress syndrome (ARDS) are common forms of hypoxic respiratory failure in patients with a mortality rate of 25–40% [1]. ALI/ARDS is also a major complication and a common cause of mortality in severely burned patients [2]. Growing evidence from experimental and clinical studies had shown that the systemic inflammatory response plays critical roles in the development and mortality of ALI/ARDS [3–5]. Several studies have shown that transplantation of MSCs can reduce the systemic inflammation, attenuate lung injury, and improve survival in models of ALI [6–8]. Unfortunately, there are no animal models of severe burn-induced ALI in these studies. The human umbilical cord MSCs (hUC-MSCs) emerged recently as an attractive source of MSCs for clinical application. The hUC-MSCs not only share the same features such as multilineage differentiation, paracrine functions, and immune-modulatory properties of all MSCs but also have some unique advantages, such as not requiring for bone marrow matching, low immunogenicity, higher self-renewal capacities, and accelerating injury tissue repair processes [9, 10]. Our previous experiments had demonstrated that severe burns could bring a systemic inflammatory reaction. Meanwhile, hUC-MSCs could alleviate the systemic inflammatory reaction of severe burn through secreting a variety of bioactive factors [11]. However, little is known about hUC-MSCs in the prevention of severe burn-induced ALI. The multipotential anti-inflammatory protein tumor necrosis factor- (TNF-*α*-) stimulated gene/protein 6 (TSG-6) has been found to be efficacious in a wide range of disease models, including respiratory disease models. Our previous study further testified the potential role and mechanism of hUC-MSCs secreting TSG-6 that attenuated severe burn-induced excessive inflammation via inhibiting activation of P38 and JNK signaling ways [11, 12]. Above discoveries prompted us to further investigate whether hUC-MSCs attenuate severe burn-induced ALI via paracrine secreting TSG-6, which inhibits the inflammatory reaction in lung tissue. Within this study, we investigated the therapeutic potential and mechanism of hUC-MSCs on severe burn-induced ALI in a rat model.

## 2. Materials and Methods

### 2.1. hUC-MSC Preparation

All experimental procedures were in accordance with the international guidelines for biomedical research in experimental animals issued by the Committee of International Organizations of Medical scientific (CIOMS). We approved the certification of the institutional animal care and use committee of the Fourth Medical Center of PLA General Hospital and obtained the informed consent of all subjects. The primary human umbilical cord mesenchymal stem cells (hUC-MSCs) were purchased from ScienCell Research Laboratories (ScienCell, San Diego, CA). The hUC-MSCs were examined using flow cytometry and are positive for CD105, CD90, CD44, CD29, and HLA-I and negative for CD34, CD45, HLA-DR, CD31, and vWF as described in our previous publication [12]. They have the potential to develop into mature cells that produce fat, cartilage, bone, tendons, and muscle. These properties, in combination with their developmental plasticity, have generated tremendous interest because of the potential use of MSC in regenerative medicine to replace damaged tissues.

### 2.2. Ex Vivo Evaluation of TSG-6 Released from hUC-MSCs in Serum of Burn Animals

The hUC-MSCs were plated into 6-well plates with a cell density of 5000 cells/cm^2^ and incubated with 3 ml mesenchymal stem cell medium serum free (MSCM-sf) (ScienCell Research Laboratories, San Diego, CA) for 1 day, which were subsequently cultured continuously with 3 ml of MSCM-sf containing 10% sham serum or burn serum at 24 hours postsevere burn. Currently, the levels of inflammatory mediators, oxidative stress factors, and other factors were very high. TSG-6 secretion from hUC-MSCs after 10% burn serum continuous culture and the supernatant of the cells were assayed by using the ELISA kit.

### 2.3. hUC-MSCs Were Transfected with siRNA for TSG-6

We selected the lentiviral expression vector containing the TSG-6 siRNA sequence (GeneChem, Shanghai, China) for specific targeting TSG-6, including Lenti-TSG-6-siRNA, and its negative control vector, Lenti-GFP-siRNA. TSG-6 siRNA lentivirus vectors were generated by ligating the vector PGC-LV. TSG-6 siRNA oligonucleotide sequences are forward 5′-CCGGTTCTCCGAACGTGTCACGTTTCAAGAGAACGTGACACGTTCGGAGAATTTTTG-3′ and reverse 5′-AATTCAAAAATTCTCCGAACGTGTCACGTTCTCTTGAAACGTGACACGTTCGGAGAA-3′.

The sequences of control siRNA are forward 5′-CCGGGCAAGCTGACCCTGAAGTTCATCTCGAGATGAACTTCAGGGTCACGTTGCTTTTTG-3′ and reverse 5′-AATTCAAAAAGCAAGCTGACCCTGAAGTTCATCTCGAGATGAACTTCAGGGTCACGTTGC-3′.

The recombinant lentivirus was produced by cotransfecting HEK293T cells with pGC-LV-GFP-siRNA or pGC-LV-TSG-6-siRNA, pHelper 1.0, and pHelper 2.0 plasmids using Lipofectamine 2000 (GeneChem, Shanghai, China). The hUC-MSCs were transfected with the prepared lentivirus (Lenti-TSG-6-siRNA or Lenti-GFP-siRNA). The hUC-MSCs were plated into either 6-well plates or T-75 culture bottles with a cell density of 5000 cells/cm^2^ and incubated with 3 ml or 10 ml MSCM-sf without antibiotics for 1 day. And then, these cells were transfected with 1 ml medium of Lenti-TSG-6-siRNA or Lenti-GFP-siRNA according to the protocol provided by the manufacturer. After 12 hours, the medium was changed, and these cells were incubated with MSCM-sf without antibiotics for 3–4 days sequentially. The efficiency of TSG-6 gene knockdown in hUC-MSCs was evaluated using GFP-fluorescence observation and Western blot. Then, hUC-MSCs in T-75 culture bottles were harvested with 0.25% trypsin and resuspended at 5 × 10^6^ cells in 1 ml sterile PBS for subsequent implantation.

### 2.4. Animals

All studies adhered to procedures consistent with the International Guiding Principles for Biomedical Research Involving Animals issued by the Council for the International Organizations of Medical Sciences (CIOMS), and all experimental protocols were approved by the Institutional Animal Care and Use Committee at the Fourth Medical Center of PLA General Hospital and were performed in accordance with the AVMA Guidelines for the Euthanasia of Animals: 2013 Edition. Six-week-old male Wistar rats (180–220 g) were obtained from the local animal facility and housed at the Institute of Animal Experiments of the Fourth Medical Center of PLA General Hospital in stables with a temperature of 22°C, a relative humidity of 55%, and a day/night cycle of 12/12 hours, with food and water ad libitum throughout the experiment.

### 2.5. Experimental Groups

Wistar rats were randomly divided into 5 groups: (1) sham, (2) burn, (3) burn+hUC-MSCs (burn and implantation with hUC-MSCs), (4) burn+vehicle (burn and implantation with hUC-MSCs transfected with Lenti-GFP-siRNA), and (5) burn+siTSG-6 (burn and implantation with hUC-MSCs transfected with Lenti-TSG-6-siRNA). Rats in each group were divided equally into three subgroups of 6 rats according to the indicated time points of d1, d3, and d7 for the samples collected.

### 2.6. Animal Treatment

The rats were anesthetized with Avertin (20 mg/ml and 300 mg/kg) (2,2,2-tribromoethanol, Sigma, USA) via intraperitoneal injection. The dorsal and abdominal hair was completely shaved. Rat models with 50% TBSA full-thickness burn injury were established via placing the backside and abdomen into hot water (94°C) for 12 s and 6 s, respectively [11, 13, 14]. Intraperitoneal injections of balanced salt solution (40 ml/kg) were immediately administered to prevent shock in all groups. We adopted a 20-gauge central catheter as a tracheal catheter, while rats were restrained on the 45° inclined operating board with their head facing the operator. The upper incisors of the rat were fixed with a cotton thread to the nails at one end of the operating board, so the head of the rat was raised, and we inserted the laryngoscope into its mouth. The glottis of the rat can be seen in the light of the laryngoscope. The tracheal catheter was quickly inserted into the airway when the glottis was opened [15, 16]. The rats in the burn+hUC-MSC group immediately received intratracheal implantation of 5 × 10^6^ (1 ml) hUC-MSCs after burn. The rats in the burn+vehicle and burn+siTSG-6 groups received intratracheal implantation of 5 × 10^6^ (1 ml) hUC-MSCs transfected with Lenti-GFP siRNA or Lenti-TSG-6-siRNA at the same time. The rats in the sham and burn groups received intratracheal implantation of 1 ml PBS. All rats in each group were clinically evaluated. The burn wound and rats in the sham group were treated as previously described [11]. The blood and lung tissue samples were collected at each indicated time point. After anesthesia with the above anesthetics, the rats' blood was extracted from the inferior vena cava. And then, rats were euthanized with an overdose of pentobarbital sodium intravenously.

At each indicated time point, blood samples from the inferior vena cava were extracted, and lung tissue samples were collected. And then, rats were euthanized with an overdose of pentobarbital sodium intravenously.

### 2.7. Specimen Collection and Detection

Both the lung tissue and blood samples in all groups were taken from the aorta ventralis at d1, d3, and d7 after injury. Subsequently, one piece of lung specimen was stored in liquid nitrogen for TSG-6 detection using the ELISA kit; also, another piece was fixed using 4% paraformaldehyde for immunohistochemical analysis and H&E staining. The detection range of the kit is from 0.312 ng/ml to 20 ng/ml. 100 mg lung tissue was rinsed with PBS, homogenized in 1 ml of PBS, and stored overnight at −20°C. After two freeze-thaw cycles were performed to break the cell membranes, the homogenates were centrifuged for 5 minutes at 5000 g at 2–8°C. The supernatant was removed and assayed immediately. The standard was diluted following the manufacturer's instruction. Use the stock solution to produce a 2-fold dilution series. Mix each tube thoroughly before the next transfer. The undiluted standard serves as the high standard (20 ng/ml). The sample diluent serves as the standard zero.

### 2.8. Histological Analyses

After fixation with 4% paraformaldehyde for 1 week at room temperature, the specimens were embedded in paraffin and sectioned into five-micrometer-thick sections. Then, the slices were deparaffinized with dimethyl benzene, rehydrated, and then stained with H&E in accordance with standard procedures. Infiltrations of neutrophils and macrophages were detected via incubating with specific antibodies (MPO, CD68; from R&D Systems, Minneapolis, MN), the corresponding secondary antibody, and the PAP (peroxidase-antiperoxidase) complex in turn and finally stained with DAB (3,3′-diaminobenzidine). Myeloperoxidase (MPO) exists in the cytoplasm of neutrophils and can be used as an indicator of neutrophils in lung tissue. The positive reaction of neutrophils is brown-yellow colorization. CD68 is a macrophage-specific antigen which is direct evidence for the identification of macrophages. The positive reaction of macrophages is brown colorization. Randomly, five different visual fields under a light microscope (400x) of each respective section were chosen, and CD86- and MPO-positive cells were counted by an experienced scientist in a blinded manner.

### 2.9. Western Blotting

Protein from hUC-MSCs was isolated using RIPA buffer (Sigma Life Science), containing PMFS (Sigma Life Science) and complete Mini Protease inhibitor cocktail (Roche). Cells were centrifuged at 3000g for 10 min to collect the nuclei, and then, the supernatants were further centrifuged at 17,000g for 20 min to separate the mitochondrion. The cytoplasm was diluted in Laemmli buffer and denaturized at 95°C for 10 min. Protein was quantified using a commercial bicinchoninic acid (BCA) kit (BCA Protein Assay Kit; Pierce Biotechnology Inc., Rockford, IL, USA). Equal amounts of protein were subjected to SDS-PAGE gel as previously described [17], and the anti-TSG-6 antibody (1 : 1,000) and anti-*β*-actin antibody (1 : 20,000) (R&D Systems, Minneapolis, MN) were used for the protein expression assay.

### 2.10. Enzyme-Linked Immunosorbent Assay (ELISA)

100 mg lung tissue was rinsed and homogenized and then stored overnight at −20°C. The samples were performed to break the cell membranes; the homogenates were centrifuged for 5 minutes at 5000*μ*g at 2–8°C. The supernatant was removed. The double-antibody sandwich ELISA kits (eBioscience, USA) were used to detect the levels of tumor necrosis factor alpha (TNF-*α*), interleukin-1*β* (IL-1*β*), IL-6, IL-10, MPO, and TSG-6 in lung tissues and serum according to the manufacturer's protocols.

### 2.11. Statistical Analysis

All data were expressed as the mean ± SD and analyzed using SPSS 18.0 (SPSS Inc., Chicago, IL, USA). ANOVA followed by the Tukey Cicchetti test for multiple comparisons or nonparametric Kruskal-Wallis test as appropriate was used for statistical analysis. Statistical significance level was set at *p* < 0.05.

## 3. Results

### 3.1. TSG-6 Levels in Supernatant/Lung Tissues/Serum and TSG-6 Knockdown Efficiency

**Figure 1:**
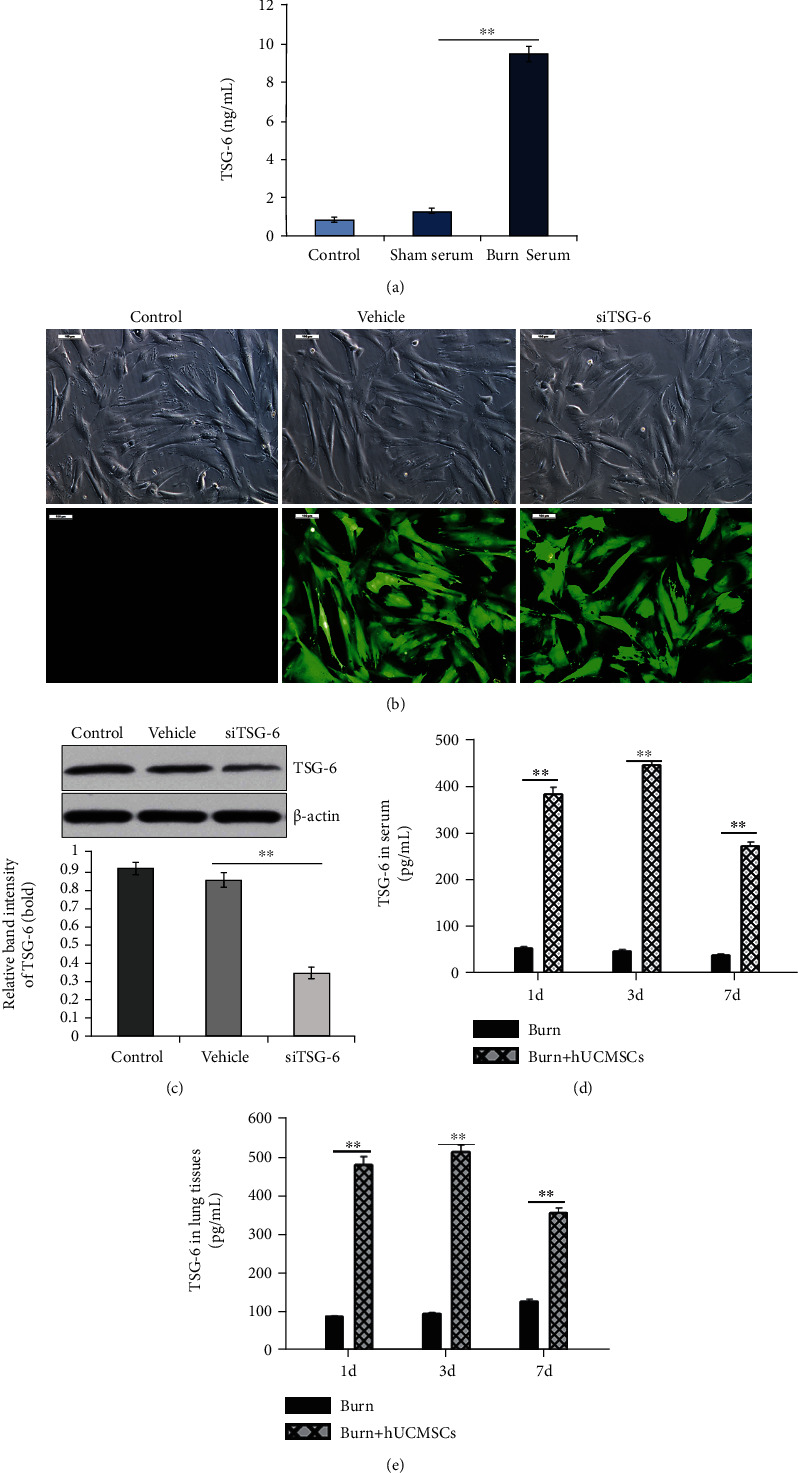
Secretion and knockdown efficiency of the TSG-6. (a) TSG-6 levels in supernatants of control, sham serum, and burn serum groups were evaluated by ELISA. Compared with control and sham groups, the TSG-6 levels in the burn serum group were significantly increased. (b) Representative photographs demonstrated successful transfection with Lenti-GFP-siRNA (vehicle) and Lenti-TSG-6-siRNA (siTSG-6) in hUC-MSCs (the inverted fluorescence microscope, 100x). (c) The levels of TSG-6 protein expression in hUC-MSCs after transfection with Lenti-GFP-siRNA and Lenti-TSG-6-siRNA were detected by Western blotting, and quantitative analysis is shown in the corresponding histogram. (d, e) TSG-6 levels in lung tissue and serum of burn and burn+hUC-MSC groups were also evaluated using ELISA. TSG-6 level of the burn+hUC-MSC group was significantly higher than that of the burn group. Values are represented as mean ± SD (*n* = 6). Asterisk (∗) and double asterisk (∗∗) stand for *p* < 0.05 and *p* < 0.01, respectively.

To investigate whether TSG-6 secreted from hUC-MSCs influenced their therapeutic effect or not, we firstly examined the levels of TSG-6 secretion from hUC-MSCs after treatment with serum of sham or burn. Compared with the sham group, TSG-6 levels in the supernatant of burn serum were significantly increased in vitro at 24 hours postburn (Figure 1(a)). The hUC-MSCs cultured in vitro were transfected with Lenti-GFP siRNA or Lenti-TSG-6-siRNA. As shown in Figure 1(b), representative photographs demonstrated successful transfection with Lenti-GFP siRNA or Lenti-TSG-6-siRNA. The data from Western blotting showed that TSG-6 expression was significantly decreased after Lenti-TSG-6-siRNA transfection compared with that of the vehicle group (Figure 1(c)). In vivo, TSG-6 levels of the burn+hUC-MSC group in lung tissues were significantly increased compared with those of the burn group at d1, d3, and d7 postsevere burn (Figure 1(d)). Meanwhile, TSG-6 levels of the burn+hUC-MSC group in serum were significantly increased compared with those in the burn group in vivo (Figure 1(e)). TSG-6 levels of lung tissues and serum reached the peak at d3 in the burn+hUC-MSC group.

### 3.2. The Effect of hUC-MSCs and TSG-6 Knockdown on Structural Damage and Total Inflammatory Cell Infiltration in Lung Tissues after Severe Burn

**Figure 2:**
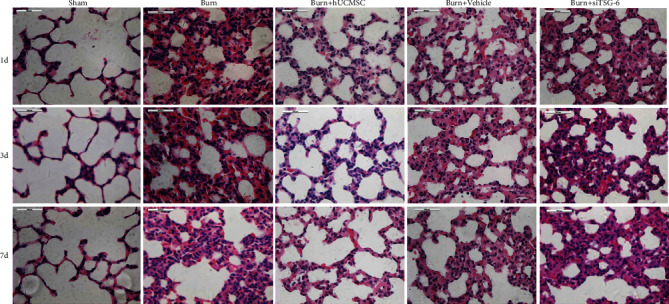
Effect of TSG-6 on the structural damage and total inflammatory cell infiltrations of lung tissues induced by severe burn. Representative photographs of lung tissues in all groups using H&E staining are shown (the light microscope, 400x).

**Figure 3:**
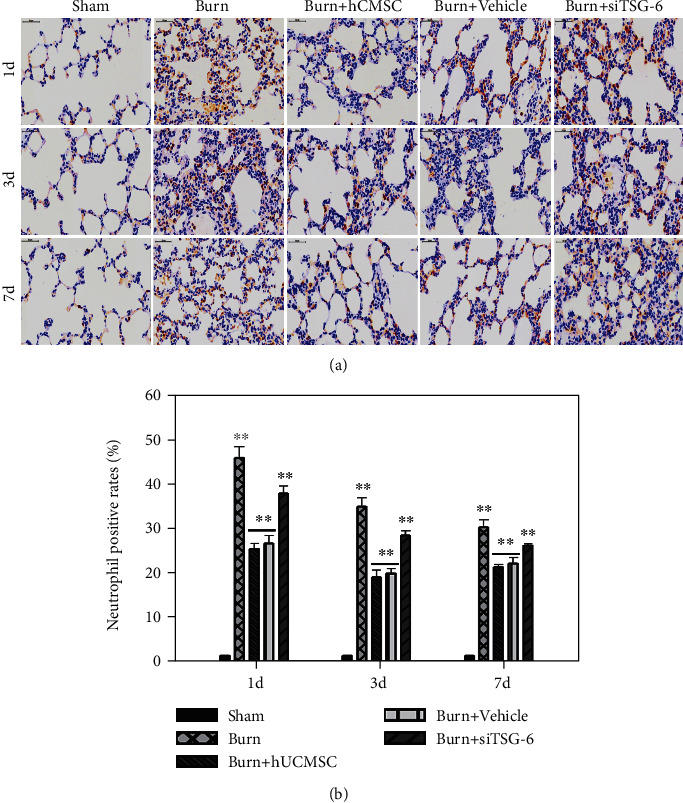
Effect of TSG-6 secretion and knockdown on neutrophil infiltrations of lung tissues induced by severe burn. (a) Representative photographs of MPO^+^ in lung tissues using immumohistochemical staining are shown (the light microscope, 400x). (b) The quantitative analysis is shown in the corresponding histogram. Values are represented as mean ± SD (*n* = 6). Asterisk (∗) and double asterisk (∗∗) stand for *p* < 0.05 and *p* < 0.01, respectively.

**Figure 4:**
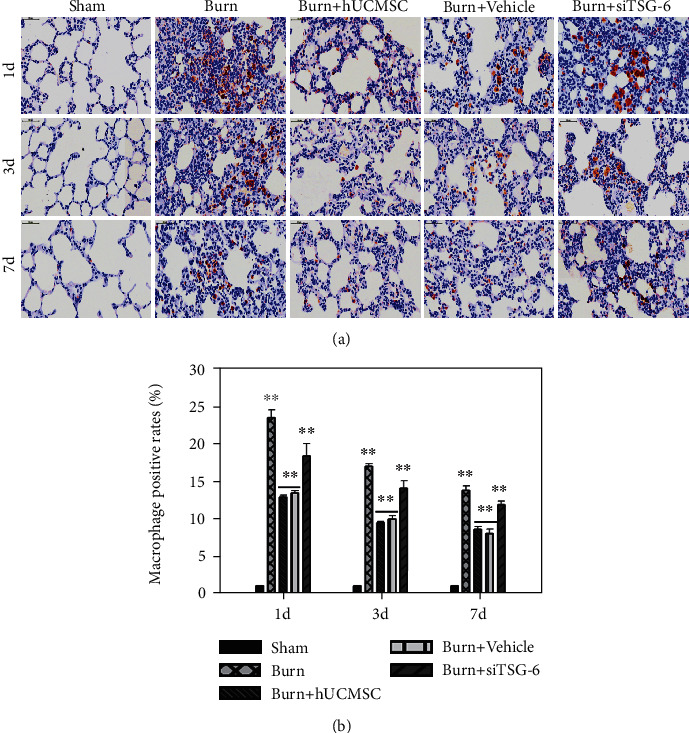
Effects of TSG-6 secretion and knockdown on macrophage infiltrations of lung tissues induced by severe burn. (a) Representative photographs of CD68^+^ in lung tissues using immumohistochemical staining are shown (the light microscope, 400x). (b) The quantitative analysis is shown in the corresponding histogram. Values are represented as mean ± SD (*n* = 6). Asterisk (∗) and double asterisk (∗∗) stand for *p* < 0.05 and *p* < 0.01, respectively.

We evaluated the therapeutic influence of hUC-MSCs and TSG-6 knockdown on the structural changes and infiltrated degree of total inflammatory cells in the lungs at d1, d3, and d7 postsevere burn using H&E staining. As shown in Figure 2, compared with the sham group, the structural damage, including alveoli fusion, interstitial hemorrhage, alveoli exudation, and infiltrated degree of total inflammatory cells in the lung tissues of the burn group, was remarkably increased at d1. Meanwhile, these changes were less severe in the burn+hUC-MSC group at d1. Destroyed structure and inflammatory cell infiltration after burn, such as neutrophil infiltration, interstitial edema, and pulmonary hemorrhage, were all improved gradually at d3 and d7. However, we noted that burn+hUC-MSCs recovered faster than other groups. Moreover, we found that the therapeutic effect of hUC-MSCs on the structural damage and total inflammation was sharply decreased after TSG-6 knockdown (Figure 2). Respectively, we calculated the neutrophil and macrophage infiltration of lung tissues induced by severe burns to evaluate the therapeutic influence of hUC-MSCs and TSG-6 knockdown on lung tissues at d1, d3, and d7 after severe burns using immunohistochemical staining. As shown in Figure 3, compared with the sham group, the neutrophil infiltration degrees of lung tissues in the burn group were remarkably increased, and they were markedly decreased by hUC-MSC administration (*p* < 0.05). Furthermore, the data showed that the therapeutic effect of hUC-MSCs on neutrophil infiltration in the lung was sharply decreased after TSG-6 knockdown (Figure 3(a)). The quantitative analysis was reported in the corresponding histogram (*p* < 0.05) (Figure 3(b)). As shown in Figure 4, in comparison with those in the sham group, macrophage infiltration degrees of lung tissues in the burn group were significantly increased, which were dramatically decreased after hUC-MSC implantation (*p* < 0.05). Furthermore, the data showed that the therapeutic effect of hUC-MSCs on macrophage infiltration in the lung was also sharply decreased after TSG-6 knockdown (Figure 4(a)). The quantitative analysis was reported in the corresponding histogram (*p* < 0.05) (Figure 4(b)). It suggested that the therapeutic effect of hUC-MSCs on attenuating burn-induced neutrophil and macrophage infiltration was dependent on the level of TSG-6 expression. Downregulation of TSG-6 expression in hUC-MSCs significantly reduced their therapeutic function on improving structural damages and total inflammatory cell infiltrations of the lung tissues (Figures 2–4).

### 3.3. The Effect of hUC-MSCs and TSG-6 Knockdown on Inflammatory Cytokines in Lung Tissues after Severe Burn

**Figure 5:**
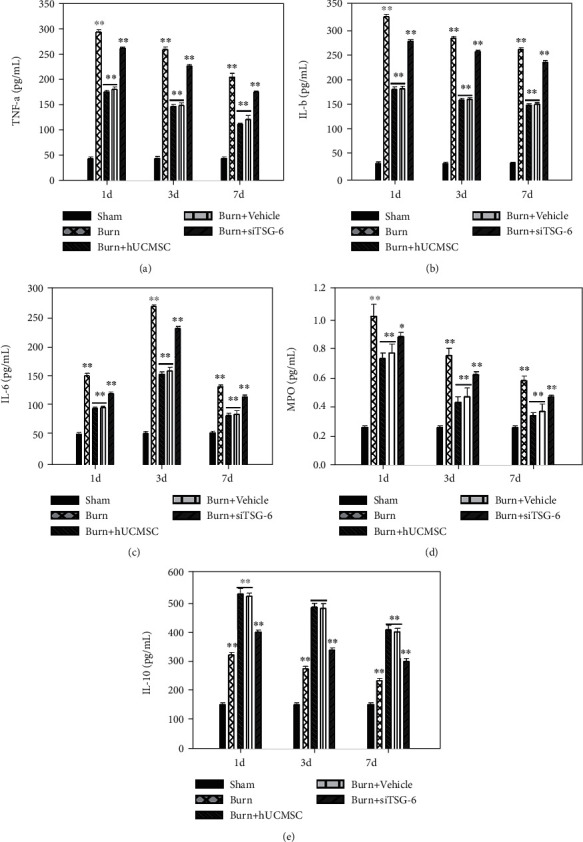
Effect of TSG-6 secretion and knockdown on inflammatory cytokines of lung tissues induced by severe burn. The effects of TSG-6 secretion and knockdown on TNF-*α* (a), IL-1*β* (b), IL-6 (c), MPO (d), and IL-10 (e) levels of lung tissue at 1 d, 3 d, and 7 d postsevere burn, respectively. Values are represented as mean ± SD (*n* = 6). Asterisk (∗) and double asterisk (∗∗) stand for *p* < 0.05 and *p* < 0.01, respectively.

We also tested the levels of the proinflammatory cytokines TNF-*α*, IL-1*β*, IL-6, and MPO and the anti-inflammatory cytokine IL-10 in lung tissues at d1, d3, and d7 after severe burn using ELISA. As shown in Figure 5, compared with the sham group, the levels of proinflammatory cytokines, such as TNF-*α* (Figure 5(a)), IL-1*β* (Figure 5(b)), IL-6 (Figure 5(c)), and MPO (Figure 5(d)), in the lung tissues of the burn group were remarkably increased, and they were all markedly decreased after hUC-MSC administration (*p* < 0.05). It is worth noting that the level of the anti-inflammatory cytokine IL-10 (Figure 5(e)) in the burn+hUC-MSC group was significantly higher than that in the burn group and sham group, and that in the burn group was also higher than that in the sham group (*p* < 0.05).

While the data also indicated that the therapeutic effect of hUC-MSC knockdown TSG-6 surely increased the levels of proinflammatory cytokines, such as TNF-*α*, IL-1*β*, IL-6, and MPO, and decreased the anti-inflammatory cytokine IL-10 in lung tissues (*p* < 0.05) (Figure 5).

## 4. Discussion

During the last few years, human MSCs as a cellular therapy for the ALI/ARDS have ignited much interest of researchers, and the therapeutic effects have been demonstrated in different animal models [18–20]. Studies have shown that MSCs have therapeutic effects in various preclinical models of lung diseases via direct differentiation and paracrine action [21–23]. Indeed, there were a few of MSCs that survive for more than one week after systemic administration, suggesting that the main effects of MSCs are probably mediated by paracrine mechanisms [24].

Patients with ALI caused only by severe burns or scald were not uncommon in the clinic, especially in children. In the present study, we established a severe burn-induced ALI in a rat model to investigate the therapeutic effects of hUC-MSCs and their possible mechanism. We found that intratracheal implantation of hUC-MSCs increased the survival rates and significantly attenuated severe burn-induced ALI via inhibiting pulmonary inflammation, and the therapeutic effects of hUC-MSCs were strongly reduced when the expression of TSG-6 was inhibited via RNA interference. It indicated that the therapeutic effects of hUC-MSCs in severe burn-induced ALI rats were associated with the soluble factor TSG-6 secreted by hUC-MSCs itself.

We created a rat model with a classical pulmonary inflammatory state via severe burn [13, 14, 25]. The severity of the lung injury was characterized by several pathological parameters, just as described in the diagram (Figures 2–4). All these pathological changes are closely tied to the human ALI, which indicates that our model successfully duplicated human ALI. These parameters were all improved after intratracheal implantation of hUC-MSCs, which indicated the therapeutic benefits of hUC-MSC implantation on severe burn-induced ALI. These findings proved that the intratracheal injection of hUC-MSCs attenuated lung injury in severe burn-induced ALI rats. Our results were consistent with some other previous studies, in which the administration of hUC-MSCs or BM-MSCs reduced systemic inflammation and attenuated LPS-induced ALI in rats [26].

ALI is an uncontrollable pulmonary inflammatory disease characterized by the release of cytokines and neutrophil accumulation. Neutrophils are the primary cells being convened to the site of inflammation and releasing inflammatory cytokines. Macrophages are another predominant cells existing in either a proinflammatory (M1) or anti-inflammatory (M2) state, acting as a trigger of the inflammatory response, and contributing to both the initiation and resolution of inflammation. MSC treatment could regulate macrophages with the anti-inflammatory phenotype and simultaneously enhance the phagocytic activity of macrophages [27]. In our study, the infiltration of neutrophils and macrophages in lung tissue in the burn group was remarkably increased and surely reduced after being treated with hUC-MSCs. This alleviative lung injury of hUC-MSCs was once confirmed in canine radiation-induced ALI model via reducing oxidative stress, inflammatory reactions, and TGF-*β*-Smad 2/3 pathway activation [28].

MPO is an enzyme stored in neutrophils and macrophages and released into the extracellular fluid in the setting of the inflammatory process. Since MPO is an important enzyme in the inflammatory process, there is an ongoing interest in the use of MPO as a biomarker for assessing the extent of inflammatory response in our present study. After a severe burn, a remarkable increase in MPO in lung tissues was detected in the burn group, and the peak level was shown at d1. As we expected, treatment with hUC-MSCs clearly reduced the MPO activity at d1, d3, and d7. Previously, Li et al. found that intravenous infusion with hUC-MSCs significantly inhibited LPS-stimulated MPO activity in rat lung tissues [26]. Reduced MPO activity indicates an improvement of lung injury and confirmed the therapeutic effects of hUC-MSCs on burn-induced lung injury through promoting anti-inflammatory homeostasis.

The proinflammatory cytokines TNF-*α*, IL-1*β*, and IL-6 are the primary mediators of the acute phase response. Previous studies showed that the decreased neutrophil recruitment into the lung tissue and suppressed expression of TNF-*α*, IL-1*β*, and IL-6 can improve the outcomes of ALI [29]. Several other studies have also shown that MSCs could decrease the plasma level of TNF-*α* and IL-1*β* through paracrine secretion [30]. Consistent with previous studies, increased levels of these inflammatory cytokines were found in our model of severe burn-induced ALI. After severe burns, the concentration of TNF-*α*, IL-1*β*, and IL-6 significantly increased. The difference is simply that the peak level occurs at different times. For TNF-*α* and IL-1*β*, the peak level appeared on d1, while the peak level of IL-6 appeared on d3. When hUC-MSCs were intratracheally implanted after burn, the concentration of proinflammatory cytokines in lung tissues decreased significantly, which was mostly in agreement with previous studies demonstrating that BM-MSCs can reduce TNF-*α* and IL-6 secretion by lung macrophages via the paracrine pathway or direct contact with host cells [30]. Our results also confirmed that hUC-MSCs were able to protect the lung from injury through reducing the inflammatory response.

Interleukin-10 (IL-10), one of the most important anti-inflammatory cytokines, is known to reduce the synthesis of proinflammatory cytokines. IL-10 treatment is supposed to be a promising therapeutic strategy to reduce lung injury [30–32]. Some argued that the progression of ALI is associated with decreased expression and secretion of IL-10 [33]. However, in our study, the IL-10 level rose in lung tissues after burn injury, and it was further markedly elevated after hUC-MSC administration. Thus, the protective effect of IL-10 in lung inflammation response, which had been well described in previous studies, might partially explain the mechanism through which hUC-MSCs exerted their therapeutic effects on severe burn-induced ALI.

According to the above findings, it can be inferred that hUC-MSC administration improved ALI by balancing the homeostasis of the cytokine network. Furthermore, we would like to know more about how hUC-MSCs did work. In our previous study, we found that the TSG-6 levels were significantly elevated in the systemic inflammatory response in burn rats. TSG-6 expression is induced as a result of an inflammatory response. Our data showed that severe burn-induced ALI could upregulate the expression of TSG-6, which was in agreement with our previous study [11]. The even higher level of TSG-6 in the burn+hUC-MSC group might relate to the TSG-6 secreted by hUC-MSCs in response to inflammatory signals. To test our hypothesis and investigate the role of TSG-6, TSG-6 knockdown was achieved via RNA interference by transfection with siRNA for TSG-6 in our ALI rat model. In the burn+siTSG-6 group, the level of TSG-6 was significantly decreased. Meanwhile, pathological changes and inflammatory response were detected in the burn+siTSG-6 group and burn+vehicle group. These results showed that the efficacies of hUC-MSC implantation, including improvement of lung function and pulmonary metabolism function, structure protection of lung tissues, reduction of inflammatory cell infiltration, suppression of proinflammatory cytokines, and promotion of expression of anti-inflammatory, were diminished by TSG-6 knockdown. Our data inferred that the anti-inflammatory properties of hUC-MSCs in severe burn-induced ALI are explained, at least in part, by the activation of hUC-MSCs to secrete TSG-6. These researches further proved that TSG-6 is an inflammation-associated secreted protein that has been implicated as having important and diverse tissue protective and anti-inflammatory properties [34]. Our results showed the observed beneficial effects of hUC-MSCs in animal models of ALI and suggest that the anti-inflammatory properties of hUC-MSCs in the lung are explained, at least in part, by the activation of hUC-MSCs to secrete TSG-6. TNF*α*-stimulated gene-6 (TSG-6), a 30 kDa protein generated by activated macrophages, modulates inflammation; however, its mechanism of action and role in the activation of macrophages were not fully understood. From our earlier report [11] and some literatures [35], we could find that TSG-6 inhibited the association of TLR4 with MyD88, thereby suppressing NF-*κ*B activation. TSG6 also prevented the expression of proinflammatory proteins (iNOS, IL-6, TNF-*α*, IL-1*β*, and CXCL1) while increasing the expression of anti-inflammatory proteins (CD206, Chi3l3, IL-4, and IL-10) in macrophages. This shift was associated with suppressed activation of proinflammatory transcription factors STAT1 and STAT3. Thus, TSG-6 functions by converting macrophages from a proinflammatory to an anti-inflammatory phenotype secondary to suppression of TLR4/NF-*κ*B signaling and STAT1 and STAT3 activation.

It was well known that delivering locally has the advantage of reaching the target organ directly with relatively small dosage, faster and stronger effectiveness, and slighter systemic adverse reactions. In a clinical setting, physicians prefer to use medicine through intratracheal administration or aerosol inhalation for patients with lung diseases, especially for severe cases. Thus, we chose the intratracheal implantation as the route of medication. While increasing the dose of drugs might increase the efficacy of the treatment, we could not exclude systemic adverse reactions and more waste of resources caused by larger doses. Taking these factors into consideration, we selected the optimal dosage of hUC-MSCs (1 × 10^6^) as suggested in our previous study [36].

In our rat model, full-thickness burn injury was established via placing the backside and abdomen into hot water, but not the chest, so ALI is caused by severe burn-induced excessive inflammation and serious metabolic disturbances rather than full-thickness escharotic burn at the back and abdomen.

Although the 50% TBSA burn was severe, we managed to avoid the death of the rat model. In the early period of this study, we performed consistent treatments as follows: subjects were kept warm and then intraperitoneal injections of balanced salt solution (40 ml/kg) were immediately administered to prevent shock. And the burn wound was then treated with a 1% tincture of iodine and kept dry to prevent infection.

To the best of our knowledge, this is the first report about the therapeutic evaluation of hUC-MSCs in severe burn-induced ALI. TSG-6 secreted from hUC-MSCs played a key role in severe burn-induced ALI via inhibiting the inflammatory reaction in lung tissue. It suggests that intratracheal implantation of hUC-MSCs is an effective treatment for a severe burn-induced ALI rat model. Meanwhile, knockdown of TSG-6 mRNA expression in hUC-MSCs did not completely abrogate the anti-inflammatory effects suggesting that some other unknown mechanisms were involved. It means that additional experiments are required to determine the relative contribution of these factors to the beneficial effects of hUC-MSCs in lung injury.

## Data Availability

All authors are sure that all data and materials as well as software application or custom code support our published claims and comply with field standards. All materials in the manuscript including figures, tables, or text passages are originated from the authors.

## Abbreviation

TNF-*α*: Tumor necrosis factor *α*
TSG-6: TNF-*α* stimulated gene/protein 6
hUC-MSC: Human umbilical cord-derived mesenchymal stem cell
ALI: Acute lung injury
ELISA: Enzyme-linked immunosorbent assay
ARDS: Acute respiratory distress syndrome
MSCM-sf: Mesenchymal stem cell medium serum-free medium
GFP: Green fluorescent protein
MPO: Myeloperoxidase
CD68: Cluster of differentiation 68
siRNA: Small interference RNA
PMSF: Phenylmethanesulfonyl fluoride
IL: Interleukin.
